# Suicide deaths by occupation skill level and educational attainment in the United States

**DOI:** 10.1093/joccuh/uiae078

**Published:** 2024-12-27

**Authors:** Erick Messias, Enoch K Azasu, Nawar Nayeem, Ping-I Lin, Richard Grucza

**Affiliations:** Department of Psychiatry and Behavioral Neuroscience, Saint Louis University School of Medicine, St Louis, MO, United States; School of Social Work, University of Buffalo, The State University of New York, Buffalo, NY, United States; Department of Psychiatry and Behavioral Neuroscience, Saint Louis University School of Medicine, St Louis, MO, United States; Department of Psychiatry and Behavioral Neuroscience, Saint Louis University School of Medicine, St Louis, MO, United States; Department of Family and Community Medicine, Saint Louis University School of Medicine, St Louis, MO, United States

**Keywords:** occupational skill, education, suicide

## Abstract

**Objectives:**

To estimate the association between suicide deaths and both occupational skill level and educational attainment in the United States.

**Methods:**

Suicide deaths, employment, education, and demographic data from the 2021 National Center for Health Statistics Multiple Cause of Death Files, for ages 18-64 with a lifetime history of employment, were combined with comparable participants from the 2020-2022 Current Population Survey. Outcomes of interest were associations between suicide death and occupational skill level, assessed as a 5-level variable, and educational attainment. Logistic regression models were used to estimate these associations before and after adjusting for educational attainment, age, and sex, in each racial/ethnic subpopulation.

**Results:**

We found a significant gradient in suicide death across occupational skill levels, with lower-skill occupations associated with higher suicide rates than higher-skill occupations. This gradient was more pronounced among non-Hispanic White (NHW) people and non-Hispanic Black (NHB) people than Hispanic people. However, upon controlling for educational attainment, this association was attenuated, indicating that the initial link was likely confounded by education.

**Conclusions:**

The results suggest that educational attainment is a more robust predictor of suicide risk than occupational skill level. Targeted interventions addressing educational and ethnic disparities within higher-risk occupational groups can mitigate suicide risk.

## Teaser

Key points

- Research indicates that individuals in lower-skill occupations face a significantly higher risk of suicide compared with those in higher-skill occupations. Educational attainment also plays a crucial role, as higher education is linked to a lower risk of suicide attempts among non-Hispanic White (NHW) people but not non-Hispanic Black (NHB) people.
- Our study reveals a clear gradient in suicide deaths across occupational skill levels, with a more significant impact observed among NHW people compared with Hispanic people. However, we found that educational attainment plays a protective role by mitigating suicide risk, highlighting the importance of addressing educational disparities.
- The relationship between occupation, education, and suicide risk differs by racial/ethnic group, suggesting the need for targeted interventions.
- These findings call for strategies to improve mental health support in lower-skill occupations, promote educational opportunities, and reduce stigma associated with low-skill jobs. Mental health professionals, policymakers, and educators can use these insights to inform preventative measures.

## 1. Introduction

Suicide is a complex and multifaceted public health concern, and exhibits a notable gradient in risk across different occupational skill levels. Research has consistently demonstrated a stepwise increase in suicide risk, with individuals in lower-skill occupations facing significantly higher risks of suicide compared with those in higher skill-level groups.^1^ This heightened risk in lower-skill occupations may be attributed to a myriad of socioeconomic disadvantages, encompassing lower levels of educational attainment, diminished income prospects, and inadequate access to essential health care services.^2^ The deleterious impact of unemployment as a significant risk factor for suicide has also been well documented in national data spanning back to the 1980s.^3^ However, very little is known about the association between suicide and occupational skill levels after educational attainment is controlled for.

In recent decades, this gradient in suicide risk has become increasingly pronounced, reflecting shifting societal dynamics and economic landscapes. A comparative analysis of suicide risk across professions in Britain highlighted a concerning trend, showing an escalation in suicide risk among manual occupations juxtaposed with a decline in risk among professional occupations from the 1980s to the early 2000s.^4^ Studies have also shown higher educational attainment is associated with a lower risk of suicide attempt among non-Hispanic White (NHW) people, but not non-Hispanic Black (NHB) people.^5^

In the United States, the exploration of these social determinants contributing to suicide mortality has gained prominence, particularly within the context of the rise in “deaths of despair.” This category encapsulates not only deaths by suicide but also fatalities stemming from drug overdoses and alcohol misuse.^6^ Despite considerable attention drawn to this phenomenon, substantial gaps persist in understanding its underlying causes, intricate mechanisms, and viable opportunities for effective intervention.^7^ Specifically, the role of certain socioeconomic factors such as educational attainment in the occupational skill–suicide link is not known with certaintly.^1^ Often associations between suicide and socioeconomic factors are estimated with either educational attainment or occupational level serving as sole proxies for socioeconomic status.^2^ Hence, it is not well understood how much of the variation in suicide driven by occupational level can be explained by educational attainment, or whether it can be explained at all. Moreover, it is also not well understood whether the entire variation in suicide is due to differences in educational attainment, which, when not controlled for, leads to spurious associations between suicide and occupational levels. In light of these pressing issues, our study delved into US mortality data to estimate the suicide rate stratified by race and occupational skill level, controlling for the influence of educational attainment. Because prior investigations have studied education and skill level separately, our study adds to the existing literature by helping to understand which variable makes a stronger contribution to risk when both are included in the model. This approach aimed to shed further light on the interplay between occupational factors, educational status, and suicide death, ultimately informing targeted interventions and policies to mitigate this public health challenge for at-risk groups.

## 2. Methods

Data on individual suicide deaths were obtained from the Multiple Cause of Death file for 2021 collected by the National Center for Health Statistics and curated by the National Bureau of Economic Research.^8^ More specifically, from the complete set of death records (public-use version), we selected observations for which suicide was listed as the underlying cause based on ICD-10 (International Classification of Diseases, Tenth Revision) codes converted to the Centers for Disease Control (CDC) 113-cause of death recodes (recodes 124 through 126). Living population data were constructed from the Current Population Survey Annual Social and Economic supplement (CPS-ASEC). Multiple years (2020-2022) of these data were combined to increase sample size. These datasets were obtained from the Integrated Public Use Microdata Series maintained by the Minnesota Population Center, who curate the data and harmonize variables across years.^9^

To create the combined dataset, records for suicide deaths from the National Center for Health Statistics (NCHS) files were appended to the CPS-ASEC data.

Weights were assigned as follows: Each suicide death record from the NCHS was assigned a weight of 1, because each record corresponds to an actual individual who died by suicide (a complete enumeration). In contrast, CPS-ASEC is a survey rather than a census, and each respondent represents a certain number of people in the US population. Therefore, ASEC records were initially assigned their original survey weights, which correspond to the population size each respondent represents. But because the combined dataset spans 3 years, the original ASEC survey weights were divided by 3 to ensure proper weighting and avoid overrepresentation of the living population relative to the death records.

Job Zone/skill level is a variable used by the Occupational Information Network.^10^ O^*^NET is a database of occupations and occupation-specific descriptors that can be linked to standardized occupation codes. The Job Zone descriptor is a 5-level variable that groups occupations with similar levels of required experience, training, or educational attainment. For example, Job Zone 5 includes physicians, scientists, and lawyers. Job Zone 1 includes landscapers, store clerks, and many other service occupations. Job Zone level for 923 distinct occupations labeled by 2018 standard occupation classification (SOC) codes were downloaded from O^*^NET.^10^ SOCs are 8-digit codes linked to specific job titles and are the federal standard for occupational data.

The NCHS mortality files contain a variable for “usual occupation” that was derived from the corresponding field on the standard death certificate. These fields were recorded (by the CDC) using the 2010 Census occupation code. CPS-ASEC data contain 2018 census codes based on respondents’ usual occupation in the past year. We converted both sources of data to 2018 SOC codes using a crosswalk obtained from the US Census Bureau.^11^ However, the crosswalk file contained 6-digit SOC codes, whereas the O*NET files contained more detailed 8-digit codes (the final 2 digits are right-of-decimal detail specifiers used only for some groups of occupations). More specifically, the 923 detailed (8-digit) codes are subsumed by 798 6-digit codes. For example, the code 17-2141 includes mechanical engineers (17-2141.00), fuel cell engineers (17-2141.01), and automotive engineers (17-2141.02). Additionally, the 6-digit SOC codes sometimes subsume 6 different SOC codes that map onto a single census code (1240) for “Other Mathematical Science Occupations.” Again, in this case, the Job Zone values for the more specific SOC codes were averaged and assigned to the less specific census code. This process resulted in the 798 6-digit census codes being reduced to 544 distinct occupation categories. Of these, 107 (20%) required averaging across Job Zone values from more detailed occupations, whereas 437 (80%) did not.

### 2.1. Inclusion and exclusion criteria

Because of our interest in occupational skill level the following were excluded from the 48 338 suicides contained in the 2021 mortality files: deaths for whom only unpaid work or no employment was reported (*n* = 6578, 13.6%), those with missing occupation data (*n* = 176, 0.36%), those reporting military occupations—as military bases are not included in CPS-ASEC sampling—(*n* = 743, 1.54%), and those not between ages 18 and 64, that is, not working age (*n* = 11 446, 23.7%). This resulted in a cohort of 29 395 suicide deaths. Applying parallel exclusion to the CPS-ASEC samples resulted in a final reference population sample of 276 226 respondents, representing 198.7 million individuals.

### 2.2. Additional variables

Covariates were necessarily limited to data available on death certificates (from which NCHS mortality data are abstracted). Aside from descriptive information about the context of the death, these are limited to basic sociodemographic variables. For covariates, we chose to include nonmodifiable attributes (age, sex, race/ethnicity) and education. The latter was included as an indicator of occupational status and so that the relative importance of education compared with occupational status could be evaluated Race/ethnicity was recoded from more detailed records in both data sources (CPS-ASEC and mortality data) as NHW people, NHB people, and Hispanic people. Other groups were not included in the analysis because of small sample sizes, and “Other” was not added as a category because of the analysis's focus on differences by race/ethnicity. Single-year age was recoded to 5-year groups, except for an “18-19” category, hence 18-19, 20-24, 24-29, etc. Educational attainment was categorized into 5 categories: no high-school diploma or graduate equivalent diploma, high-school diploma only, some post-secondary education but no 4-year degree, 4-year/bachelor’s degree or higher, and advanced degree.

### 2.3. Statistical analysis

Analyses were conducted in SAS version 9.4. Descriptive statistics were tabulated separately for the suicide cohort and the CPS-ASEC living population controls. For the latter, both weighted and unweighted descriptive statistics are described. Due to the large sample size, all differences between the cohort of suicide cases and living population controls are statistically significant. Therefore, the standardized mean difference (SMD) is reported as a measure of the magnitude of the difference rather than reporting *P* values. SMDs reflect differences between the cases and the weighted control cohort.

**Table 1:** Number of suicide deaths and baseline population for skill level and educational attainment.^a^

**Characteristic** ^**b**^	**Suicide deaths**	**CPS-ASEC population (unweighted)**	**CPS-ASEC population (weighted)**	**SMD** ^**c**^
**Age**	41.6 (±12.9)	41.0 (±12.6)	40.7 (±12.9)	0.07
**Sex**				
**Male**	22 539 (81.7)	107 042 (52.9)	78 658 011 (53.6)	0.63
**Female**	5045 (18.3)	95 426 (47.1)	68 034 860 (46.4)	−0.63
**Race/ethnicity**				
**Non-Hispanic White**	21 866 (79.3)	133 475 (65.9)	97 009 688 (66.1)	0.30
**Non-Hispanic Black**	2409 (8.7)	23 799 (11.8)	19 287 779 (13.2)	−0.15
**Hispanic**	3309 (12.0)	45 194 (22.3)	30 395 405 (20.7)	−0.23
**Educational attainment**				
**<Diploma**	3173 (11.5)	15 792 (7.8)	10 740 415 (7.3)	0.14
**Diploma/GED**	12 301 (44.6)	55 643 (27.5)	40 347 766 (27.5)	0.36
**Some post-secondary**	7025 (25.5)	57 150 (28.2)	41 157 256 (28.1)	0.06
**4-Year degree**	3545 (12.9)	47 599 (23.5)	35 785 067 (24.4)	−0.30
**Advanced degree**	1540 (5.6)	26 284 (13.0)	18 662 366 (12.7)	−0.25
**Job Zone/skill level** ^**d**^				
**1 (little/no prep)**	1171 (4.3)	9496 (4.7)	6 627 257 (4.5)	0.21
**2 (some prep)**	13 606 (49.3)	83 850 (41.4)	61 464 401 (41.9)	0.50
**3 (medium prep)**	5901 (21.4)	32 830 (16.2)	23 538 051 (16.0)	0.39
**4 (considerable prep)**	5785 (21.0)	61 341 (30.3)	44 678 830 (30.5)	−0.44
**5 (extensive prep)**	1121 (4.1)	14 951 (7.4)	10 384 331 (7.1)	−0.23
**Total**	**27 584**	**202 468**	**146 692 871**	

Abbreviations: CPS-ASEC, Current Population Survey Annual Social and Economic supplement; GED, General Educational Development; SMD, standardized mean difference.

^a^Due to the large sample size, differences in the covariate proportions between cases and controls are all highly significant.

^b^Data are *n* (%) or mean (±SD).

^c^The SMD is listed in lieu of *P* values as a measure of the magnitude of the difference.

^d^A Job Zone in the O^*^NET (Occupational Information Network)^10^ system is a 5-level classification system that groups various occupations based on the level of education, experience, and training typically required for entry into the job. The 5 levels, going from 1 to 5, represent the degree of preparation needed for different types of jobs.

We planned 2 series of logistic regression models: the first to examine the association between suicide death and occupational status operationalized as Job Zone, and the second to assess the degree to which any such association remained after controlling for educational attainment. Due to known differences in associations between age and suicide risk by race,^12^ models were estimated separately for each of the 3 major race/ethnicity groups. Thus, the first set of models included Job Zone as the primary predictor with controlling for age and sex included as covariates, and stratifying by race/ethnicity group. A second set of models was estimated that also adjusted for educational attainment. Logistic regression analyses were conducted using “proc surveylogistic,” which uses the Taylor method for variance estimation in complex designs.

## 3. Results

Table 1 encompasses age, sex, race/ethnicity, educational attainment, and Job Zone. Unadjusted suicide rates for each demographic and Job Zone/skill level category, stratified by race/ethnicity, are provided in Table S1 and provide additional detail about variation in suicide death across covariates. Due to the large sample size, all differences between cases and controls are highly significant. Therefore, SMD values are provided instead of *P* values as measures of the magnitude of differences. Noteworthy differences are observed across these variables, as indicated by the SMD. For instance, in terms of sex, 81.7% of suicide deaths were male, compared with the living (employed) population, which was 53.6% male (SMD = 0.63). Suicide death was much more common among NHW people compared with either minority population. Substantial gradients were observed across both the educational attainment and Job Zone variables, with higher educational attainment and job/skill level exhibiting lower risk of suicide.

Table 2 shows the results of the regression model that examined the relationship between suicide death and Job Zone/skill level, while adjusting for age and sex but not educational attainment. Results are stratified by race/ethnicity due to anticipated differences in covariate associations with suicide across the 3 groups. Among NHW individuals, those on skill levels 1 to 4 demonstrated significantly higher odds for suicide death compared with NHW individuals on skill level 5, with females displaying significantly lower odds than males across all age groups above 18-25 years old, each showing significantly increased odds. The specific odds ratio (OR) values with 95% CIs are provided: Skill Level 1: OR = 2.35 (95% CI, 2.12-2.62), Skill Level 2: OR = 1.72 (95% CI, 1.22-2.42), and Skill Level 3: OR = 1.12 (95% CI, 0.85-1.47). Similarly, for NHB individuals, lower skill levels were associated with significantly higher odds for suicide compared with those on skill level 5. Females exhibited lower odds than males, and age groups above 18-25 years old (excluding ages 26-35) exhibited a significant decrease in odds for suicide death. NHB individuals on Skill Level 1 (OR = 2.29; 95% CI, 1.98-2.66) and Skill Level 2 (OR = 1.72; 95 l% CI, 1.22-2.42) had higher odds of suicide than those on Skill Level 5. In contrast to the non-Hispanic groups, among Hispanic individuals, compared with individuals at Skill Level 5, only individuals at Skill Level 3 demonstrated significantly increased odds (OR = 1.39; 95% CI, 1.08-1.79). Hispanic females exhibited significantly lower odds of suicide than Hispanic males (OR = 0.25; 95% CI, 0.23-0.25), and older age was associated with significantly lower odds for suicide death.

**Table 2:** Suicide deaths by training/skill level adjusted for age and sex only.

	**White non-Hispanic**			**Black non-Hispanic**			**Hispanic**		
	**OR**	**95% CI**	***P* value**	**OR**	**95% CI**	***P* value**	**OR**	**95% CI**	***P* value**
**Job Zone/skill level** ^**a**^									
**1 (little/no prep)**	2.35	2.12-2.62	<.01	1.72	1.22-2.42	<.01	1.12	0.85-1.47	n.s.
**2 (some prep)**	2.29	2.13-2.46	<.01	1.89	1.44-2.48	<.01	1.23	0.96-1.57	n.s.
**3 (medium prep)**	2.41	2.24-2.60	<.01	2.14	1.61-2.86	<.01	1.39	1.08-1.79	<.01
**4 (considerable prep)**	1.13	1.05-1.22	<.01	1.45	1.09-1.93	<.01	1.13	0.87-1.46	n.s.
**5 (extensive prep)**	1.00	ref		1.00	ref		1.00	ref	
**Age, y**									
**18-25**	1.00	ref		1.00	ref		1.00	ref	
**26-35**	1.42	1.34-1.51	<.01	0.80	0.71-0.80	<.01	1.02	0.92-1.14	n.s.
**36-45**	1.53	1.44-1.62	<.01	0.65	0.57-0.65	<.01	0.72	0.65-0.81	<.01
**46-55**	1.64	1.55-1.74	<.01	0.46	0.40-0.46	<.01	0.59	0.52-0.66	<.01
**56-65**	1.73	1.63-1.83	<.01	0.36	0.30-0.36	<.01	0.61	0.53-0.70	<.01
**Sex**									
**Female**	0.31	0.29-0.32	<.01	0.21	0.19-0.21	<.01	0.25	0.23-0.25	<.01
**Male**	1.00	ref		1.00	ref		1.00	ref	

Abbreviations: n.s., nonsignificant; OR, odds ratio; ref, reference.

^a^A Job Zone in the O^*^NET (Occupational Information Network)^10^ system is a 5-level classification system that groups various occupations based on the level of education, experience, and training typically required for entry into the job. The 5 levels, going from 1 to 5, represent the degree of preparation needed for different types of jobs.

Table 3 shows the results of the regression models that examined the relationship between suicide death and Job Zone/skill level adjusted for age, sex, and educational attainment, stratified by race/ethnicity. Across racial/ethnic groups, the association between skill level and suicide death varied. Among NHW individuals, the association between skill level and suicide death was substantially attenuated after adjusting for educational attainment, with Job Zone 3 exhibiting a modestly elevated OR, and Job Zone 4 exhibiting a slightly lower OR compared with the highest skill level group; ORs for groups 1 and 2 were not statistically significant. In contrast, there was a strong association with educational attainment, with lower educational levels exhibiting successively higher death rates than the group with advanced degrees; individuals with less than a high school education exhibited elevated odds of suicide death (OR = 5.50; 95% CI, 5.03-6.01). Age emerged as a significant factor across all races/ethnicities. Similarly, NHB individuals did not display a consistent trend in suicide death by skill level (all ORs nonsignificant): those with less than a high school education had an OR of 5.11 (95% CI, 3.85-5.11), and those at other levels of educational attainment exhibited similar risk to NHW groups. Females consistently exhibited significantly lower odds compared with males (eg, NHB females: OR = 0.23; 95% CI, 0.20-0.23). Among Hispanic individuals, Skill Levels 1 and 2 were associated with significantly lower odds of suicide death compared with Skill Level 1. Age remained a factor, and females consistently exhibited lower odds than males among Hispanic people (eg, OR = 0.27; 95% CI, 0.24-0.27).

**Table 3:** Suicide deaths by training/skill level adjusted for age, sex, and educational attainment only.

	**White non-Hispanic**			**Black non-Hispanic**			**Hispanic**		
	**OR**	**95% CI**	***P* value**	**OR**	**95% CI**	***P* value**	**OR**	**95% CI**	***P* value**
**Job Zone/skill level** ^**a**^									
**1 (little/no prep)**	0.95	0.85-1.06	n.s.	0.78	0.54-1.13	n.s.	0.56	0.41-0.76	<.01
**2 (some prep)**	0.99	0.91-1.08	n.s.	0.93	0.68-1.25	n.s.	0.66	0.50-0.87	<.01
**3 (medium prep)**	1.21	1.12-1.32	<.01	1.22	0.89-1.67	n.s.	0.84	0.63-1.11	n.s.
**4 (considerable prep)**	0.82	0.76-0.89	<.01	1.16	0.86-1.57	n.s.	0.88	0.67-1.16	n.s.
**5 (extensive prep)**	1.00	ref		1.00	ref		1.00	ref	
**Educational attainment**									
**Less than high school**	5.50	5.03-6.01	<.01	5.11	3.85-5.11	<.01	2.60	2.03-3.32	<.01
**High school**	3.61	3.36-3.87	<.01	3.39	2.63-3.39	<.01	2.47	1.97-3.13	<.01
**Some college**	2.07	1.93-2.23	<.01	2.05	1.59-2.05	<.01	1.66	1.30-2.12	<.01
**Degree**	1.21	1.12-1.30	<.01	1.07	0.82-1.07	n.s.	0.92	0.71-1.19	n.s.
**Advanced degree**	1.00	ref		1.00	ref		1.00	Ref	
**Age, y**									
**18-25**	1.00	ref		1.00	ref		1.00	ref	
**26-35**	1.60	1.51-1.70	<.01	0.87	0.76-0.87	<.05	1.05	0.95-1.16	n.s.
**36-45**	1.67	1.58-1.77	<.01	0.71	0.62-0.71	<.01	0.70	0.63-0.79	<.01
**46-55**	1.74	1.64-1.85	<.01	0.50	0.43-0.50	<.01	0.57	0.50-0.64	<.01
**56-65**	1.76	1.66-1.87	<.01	0.37	0.31-0.37	<.01	0.59	0.51-0.69	<.01
**Sex**									
**Female**	0.31	0.30-0.32	<.01	0.23	0.20-0.23	<.01	0.27	0.24-0.27	<.01
**Male**	1.00	ref		1.00	ref		1.00	ref	

Abbreviations: n.s., nonsignificant; OR, odds ratio; ref, reference.

^a^A Job Zone in the O^*^NET (Occupational Information Network)^10^ system is a 5-level classification system that groups various occupations based on the level of education, experience, and training typically required for entry into the job. The 5 levels, going from 1 to 5, represent the degree of preparation needed for different types of jobs.

## 4. Discussion

Our analyses found several significant differences in suicide death as it relates to occupational skill level and educational attainment. First, there was a significant increase in suicide death from the highest to the lowest skill level occupation that is more pronounced among NHW people, followed by NHB people, but nonexistent among Hispanic people. Second, most of the gradient in suicide death by occupational skill level is explained by educational attainment among NHW people. Third, among NHB people after controlling for educational attainment, the effect of occupational skill level on suicide death disappears. Fourth, among Hispanic people after controlling for educational attainment level the effect of occupational skill level on suicide death reverses and there is a protective effect for the 2 lowest occupational skill levels.

Research consistently shows a link between occupation and suicide death, with lower-skilled workers at a higher risk.^1^^,^^13^ This is particularly relevant for elderly white men, who have some of the highest suicide rates in the United States.^14^^,^^15^ Studies have sought to explain this using the concept of racialized economic threat, which is a pervasive ideology that amplifies the effects of status loss.^16^ This term is represented by the White employment-to-population ratio, and the study found decreasing employment to be associated with increasing suicide among the White population.^16^

Consistent with previous literature, higher educational attainment is associated with a lower risk of suicide attempt among NHW people and NHB people based on our findings.^5^ In contrast to our findings on suicide deaths, a study on suicide acceptability among African American males showed that each year of education increases the odds of suicide acceptability.^17^ The lowest suicide rates are observed among those with at least a college degree, with a consistent education gradient in suicide mortality.^18^ Furthermore, among NHW people, higher educational attainment is associated with a lower risk of suicide attempt.^5^

Our findings also suggest that there may be a protective effect on suicide for the lowest occupational skill levels among Hispanic people after adjusting for educational attainment. Suicide death rates among Hispanic people have been lower than those of NHW people, for instance, and highly influenced by sociocultural factors.^19^^,^^20^ Studies have found that cultural inhibitory factors may decrease suicidal behavior in the Hispanic population.^21^ This discrepancy may also be due to factors such as educational attainment, and studies have shown that positive relationships with adults in school and post–high school education plans were protective factors for suicide among Hispanic teens.^22^ Indeed, studies have found that low job control and certain occupations, such as elementary professions, were associated with an increased risk of suicide, indicating that the protective effect may be related to the specific work environment and occupational skill level.^1^^,^^23^ However, more research is needed to fully understand these complex relationships among Hispanic people. Also, the specific occupations that contribute to this risk among Hispanic people, and the potential role of culture-related stressors, remain areas for further investigation.^24^

These findings underscore the importance of considering the intersection of race/ethnicity, occupation, educational attainment, and other social determinants in understanding suicide risk.

## 5. Implications

The study's implications for suicide prevention policy are multifaceted. First, they emphasize the necessity of targeted interventions tailored to at-risk occupations, particularly focusing on reducing socioeconomic disparities such as limited access to health care, lower educational attainment, and income discrepancies among NHW people. Implementing mental health support programs and awareness campaigns within these vulnerable professions is crucial to mitigate the observed increase in suicide risk.

Additionally, the study underscores the pivotal role of educational attainment as a protective factor against suicide death. Policies aimed at improving access to education and addressing educational disparities are vital components of comprehensive suicide prevention strategies. Encouraging youth to stay in school longer not only promotes educational attainment but also serves as a proxy for fostering social support networks, thereby further enhancing mental health outcomes and reducing suicide death. In some countries, promoting skill and vocational schools as viable alternatives to traditional college degrees has been successful in providing economic opportunities and reducing disparities.^25^^,^^26^ Encouraging a diverse range of career paths and providing support for individuals in lower-skill occupations can contribute to improved mental health outcomes and reduced suicide risk.

## 6. Limitations

Although our data combine a large sample of individuals from the CPS-ASEC, a complete census of suicide decedents in 2021, and is enhanced by linkage of occupational codes to external data on skill level, there are important limitations that need to be considered. First, individuals with no “usual occupation” on their death record were necessarily excluded from the study. However, if these individuals had been previously employed and lost their employment, this could lead to increased suicide risk and bias the estimates if these missing observations were concentrated among certain sectors. Relatedly, because education and employment data are obtained from next of kin for decedents, there is uncertainty associated with these variables, the magnitude of which could vary by socioeconomic status, which could in turn relate to suicide risk. It is difficult to speculate about the magnitude and direction of these sources of error. Finally, a recent strand of literature has highlighted that higher suicide rates are observed in certain “blue collar” jobs with high rates of physical injuries.^27^ However, in our analysis we were not able to control for physical injuries that might be correlated with certain types of occupation levels.

## 7. Conclusion

In conclusion, our study underscores the critical role of educational attainment in mitigating suicide risk, even after accounting for occupational skill level. The findings suggest that educational attainment serves as a more significant protective factor against suicide death than skill level alone. Therefore, policies and interventions aimed at promoting educational opportunities and addressing educational disparities are crucial in reducing suicide rates, particularly among vulnerable populations. Furthermore, our study emphasizes the importance of destigmatizing lower-skill occupations and recognizing them as valid choices for individuals. Creating pathways for career advancement, increasing access to health care and mental health resources, and addressing socioeconomic inequalities are essential.

## Supplementary Material

Web_Material_uiae078

## Data Availability

This study used publicly available deidentified data from the Multiple Cause of Death file for 2021 collected by the National Center for Health Statistics and curated by the National Bureau of Economic Research (https://www.nber.org/research/data/mortality-data-vital-statistics-nchs-multiple-cause-death-data), the Integrated Public Use Microdata Series maintained by the Minnesota Population Center (https://doi.org/10.18128/D030.V10.0), and O^*^NET Online (https://www.onetonline.org/find/zone?z=0).
