# Supplementary material for: Suicide deaths by occupation skill level and educational attainment in the United States
Source: J Occup Health. 2024 Dec 27;67(1):uiae078. doi: 10.1093/joccuh/uiae078 (PMC11827085; doi:10.1093/joccuh/uiae078)
Supplement: Web_Material_uiae078 [file web_material_uiae078.zip › Supplemental Materials.docx]

**Supplemental Materials**

**Supplemental Table 1:** Unadjusted Suicide Rates per 100,000 by Job Zone/Skill level, educational attainment, Age, Sex and stratified by race/ethnicity.

|  | **White Non-Hispanic** | |  | **Black Non-Hispanic** | |  | **Hispanics** | |
| --- | --- | --- | --- | --- | --- | --- | --- | --- |
|  | **Rate** | **95% CI** |  | **Rate** | **95% CI** |  | **Rate** | **95% CI** |
| **Job Zone/Skill Level** |  |  |  |  |  |  |  |  |
| 1 | 25.8 | 23.8 - 27.8 |  | 12.9 | 10.3 - 15.5 |  | 9.1 | 7.9 - 10.4 |
| 2 | 29.2 | 28.6 - 29.9 |  | 15 | 14.2 - 15.9 |  | 11.5 | 11.0 - 12.1 |
| 3 | 30.9 | 29.9 - 31.9 |  | 12.9 | 11.5 - 14.2 |  | 12.6 | 11.5 - 13.7 |
| 4 | 14.1 | 13.7 - 14.6 |  | 8.6 | 7.7 - 9.5 |  | 9.0 | 8.2 - 9.8 |
| 5 | 12.0 | 11.2 - 12.8 |  | 5.2 | 3.9 - 6.6 |  | 7.2 | 5.5 - 8.9 |
|  |  |  |  |  |  |  |  |  |
| **Educational attainment** |  |  |  |  |  |  |  |  |
| Less than High school | 58.8 | 55.6 - 62.0 |  | 27.5 | 23.9 - 31.2 |  | 13.5 | 12.5 - 14.5 |
| High school | 40.4 | 39.4 - 41.3 |  | 18.9 | 17.7 - 20.1 |  | 14.1 | 13.4 - 14.9 |
| Some college | 20.8 | 20.2 - 21.4 |  | 10.6 | 9.7 - 11.4 |  | 9.1 | 8.4 - 9.8 |
| Degree | 11.3 | 10.9 - 11.7 |  | 5.5 | 4.8 - 6.3 |  | 5.2 | 4.5 - 5.8 |
| Advanced degree | 9.2 | 8.6 - 9.7 |  | 4.2 | 3.3 - 5.1 |  | 5.5 | 4.4 - 6.6 |
| **Age** |  |  |  |  |  |  |  |  |
| 18-25 | 17.0 | 16.3 - 17.8 |  | 19.5 | 17.7 - 21.2 |  | 13.0 | 12.0 - 14.0 |
| 26-35 | 21.6 | 20.9 - 22.2 |  | 15.3 | 14.1 - 16.4 |  | 13.8 | 13.0 - 14.7 |
| 36-45 | 22.6 | 21.9 - 23.3 |  | 11.8 | 10.7 - 12.8 |  | 9.9 | 9.2 - 10.7 |
| 46-55 | 24.2 | 23.5 - 25.0 |  | 8.4 | 7.5 - 9.4 |  | 7.9 | 7.1 - 8.6 |
| 56-65 | 25.6 | 24.8 - 26.5 |  | 6.7 | 5.7 - 7.6 |  | 8.2 | 7.2 - 9.2 |
|  |  |  |  |  |  |  |  |  |
| **Sex** |  |  |  |  |  |  |  |  |
| Female | 9.1 | 8.8 - 9.4 |  | 4.4 | 4.0 - 4.8 |  | 4.0 | 3.6 - 4.3 |
| Male | 34.2 | 33.6 - 34.7 |  | 21.3 | 20.2 - 22.3 |  | 16.1 | 15.4 - 16.7 |
|  |  |  |  |  |  |  |  |  |
| **Overall** | 22.5 | 22.2 – 22.9 |  | 12.5 | 12.0 – 13.0 |  | 10.9 | 10.5 – 11.3 |

**Supplemental Table 2:** Suicide risk by educational attainment, adjusted for age and sex only

|  | **White non-Hispanic** | | |  | **Black non-Hispanic** | | |  | **Hispanic** | | |
| --- | --- | --- | --- | --- | --- | --- | --- | --- | --- | --- | --- |
|  | **OR** | **95% CI** | **p-value** |  | **OR** | **95% CI** | **p-value** |  | **OR** | **95% CI** | **p-value** |
| **Educational attainment** |  |  |  |  |  |  |  |  |  |  |  |
| Less than High school | 5.96 | 5.50 - 6.47 | <0.01 |  | 4.53 | 3.49 - 4.53 | <0.01 |  | 1.99 | 1.60 - 2.48 | <0.01 |
| High school | 3.93 | 3.70 - 4.19 | <0.01 |  | 3.08 | 2.45 - 3.08 | <0.01 |  | 1.99 | 1.61 - 2.46 | <0.01 |
| Some college | 2.25 | 2.11 - 2.40 | <0.01 |  | 1.93 | 1.53 - 1.93 | <0.01 |  | 1.41 | 1.13 - 1.76 | <0.01 |
| Degree | 1.20 | 1.12 - 1.29 | <0.01 |  | 1.07 | 0.83 - 1.07 | <0.01 |  | 0.84 | 0.66 - 1.08 | n.s. |
| Advanced degree | 1.00 | ref |  |  | 1.00 | ref |  |  | 1.00 | ref |  |
|  |  |  |  |  |  |  |  |  |  |  |  |
| **Age** |  |  |  |  |  |  |  |  |  |  |  |
| 18-25 | 1.00 | ref |  |  | 1.00 | ref |  |  | 1.00 | ref |  |
| 26-35 | 1.61 | 1.52 - 1.71 | <0.01 |  | 0.90 | 0.79 - 0.90 | <0.01 |  | 1.09 | 0.98 - 1.21 | n.s. |
| 36-45 | 1.68 | 1.58 - 1.78 | <0.01 |  | 0.75 | 0.65 - 0.75 | <0.01 |  | 0.73 | 0.66 - 0.82 | <0.01 |
| 46-55 | 1.74 | 1.65 - 1.85 | <0.01 |  | 0.53 | 0.45 - 0.53 | <0.01 |  | 0.59 | 0.52 - 0.67 | <0.01 |
| 56-65 | 1.76 | 1.66 - 1.86 | <0.01 |  | 0.39 | 0.33 - 0.39 | <0.01 |  | 0.61 | 0.53 - 0.71 | <0.01 |
|  |  |  |  |  |  |  |  |  |  |  |  |
| **Sex** |  |  |  |  |  |  |  |  |  |  |  |
| Female | 0.31 | 0.29 - 0.32 | <0.01 |  | 0.23 | 0.21 - 0.23 | <0.01 |  | 0.27 | 0.24 - 0.29 | <0.01 |
| Male | 1.00 | ref |  |  | 1.00 | ref |  |  | 1.00 | ref |  |
